# Hemoparasites Do Not Affect Life-History Traits and Cellular Immune Response in Treefrog Hosts *Boana cordobae*

**DOI:** 10.3390/ani13223566

**Published:** 2023-11-18

**Authors:** Favio Pollo, Zulma Salinas, Mariana Baraquet, Manuel A. Otero, Pablo R. Grenat, Nancy Salas, Adolfo L. Martino, Ulrich Sinsch

**Affiliations:** 1 Ecología, Departamento de Ciencias Naturales, Facultad de Ciencias Exactas, Físico-Químicas y Naturales, Universidad Nacional de Río Cuarto, Ruta Nacional N° 36–km 601, Río Cuarto X5804BYA, Argentina; fpollo@exa.unrc.edu.ar (F.P.); zsalinas@exa.unrc.edu.ar (Z.S.); mbaraquet@exa.unrc.edu.ar (M.B.); motero@exa.unrc.edu.ar (M.A.O.); grenat@exa.unrc.edu.ar (P.R.G.); nsalas@exa.unrc.edu.ar (N.S.); amartino@exa.unrc.edu.ar (A.L.M.); 2 Instituto de Ciencias de la Tierra, Biodiversidad y Ambiente, Consejo Nacional de Investigaciones Científicas y Técnicas, Rosario S2000EZ, Argentina; 3 Department of Biology, Zoology Group, University of Koblenz, 56070 Koblenz, Germany

**Keywords:** extracellular and intraerythrocytic hemoparasites, *Dactylosoma*, *Trypanosoma*, life-history traits, leukocyte profile

## Abstract

**Simple Summary:**

Unicellular blood parasites are common in amphibians all over the world. It is poorly understood whether the hemoparasite load can cause disease or affect life-history traits. Our study on the neotropical treefrog Boana cordobae from Argentina quantified the hemoparasite load in 37 individuals and focused on their cellular immune response (leukocyte profile) and the potential effects on the life-history traits size, body condition, and age. Thirty frogs were infected by either hemogregarines or trypanosomes or both in high intensities, a prevalence unprecedented in other anuran hosts. Yet, none of them showed externally visible signs of disease or aberrant behavior. The leukocyte profile did not differ significantly between parasite-free controls and infected frogs. Age-adjusted size, body condition, and age were not affected significantly by hemoparasite load, but most parasite-free frogs were young first-breeders, suggesting that infections take place after sexual maturation of the hosts. Vectors transmitting trypanosomes and/or hemogregarines to B. cordobae remain to be identified and studied in detail.

**Abstract:**

We provide the first evidence for hemoparasites in the endemic Cordoba treefrog *Boana cordobae*. We collected 37 adult frogs at 1200 m a.s.l. in the Comechingones Mountains in the Córdoba province (Argentina). Each individual was sexed, then snout–vent length and body mass were recorded, a toe was collected for skeletochronological age determination, and a slide with a blood smear was prepared for hemoparasite screening, before releasing the frogs in situ. A total of 81% (n = 30) of the frogs were infected by hemogregarines and trypanosomes with a high intensity of infections. *Dactylosoma* was found for the first time in Argentina. Hemoparasites had no significant effect on the leukocyte profile, which we assessed from the May–Grünwald–Giemsa-stained blood smears. The neutrophils/lymphocytes ratio, indicative of stress, was insignificantly higher (0.06) in parasitized frogs than in parasite-free individuals (0.04). Infected frogs were larger than the controls, but this effect vanished when correcting size data for age. Young frogs (first-breeders) dominated the age distribution of parasite-free individuals, suggesting that infection of frogs takes usually place after sexual maturation. Vectors transmitting hemoparasites to *B. cordobae* remain to be identified. We demonstrate that moderate to high intensities of hemoparasites do not significantly affect the cellular immune response of *B. cordobae*, or any of the life-history traits studied, nor did they show any external sign of disease.

## 1. Introduction

In the current biodiversity crisis, amphibians are one of the most threatened taxa, and populations of many species are declining rapidly [1,2]. The major threats among them are habitat loss and infectious diseases, including parasitism [3]. Fungal infections such as chytridiomycosis have gained global attention, but viruses, protozoan, and metazoan parasites may negatively affect life-history traits as well [4,5,6,7,8]. Yet, hemoparasites have received little attention [9], though anurans host a wide variety of hemoparasites, such as protozoans, microfilariae, nematodes, rickettsians, and viral inclusions [10,11,12,13,14,15,16]. The impact of blood parasites on the life-history traits of amphibian hosts is poorly known but there is an agreement that pathological effects are rare [12,17,18,19,20,21]. Trypanosomes are common hemoparasites of amphibians, but only a few species are considered to harm their host significantly [12,22,23]. In trypanosomiasis, clinical signs include anorexia, lethargy, pallor, splenomegaly, and splenic necrosis, occasionally leading to death [23,24]. The life cycles of trypanosomes is heteroxenous and includes often insects as vector species. The prevalence of trypanosomes is often considerably greater in males than in females because frog-biting midges, the vectors of some trypanosomes, are attracted by the males’ advertisement calls [25,26,27]. Intracellular parasites, like hemogregarines, have also been detected within the erythrocytes of many anuran species [17,18,28,29,30]. In natural amphibian hosts, clinical diseases caused by hemogregarines, such as anemia, have not been demonstrated so far [20,31]. On a subclinical level, acute or permanent stress reactions in the presence of hemoparasites resulting in characteristic ratio alterations of the leukocyte profile have not been reported yet for amphibians [32,33,34].

In vertebrates, one line of defense against pathogens such as hemoparasites are leukocytes [35,36]. Consequently, leukocyte profiles assessed in wild populations cannot only provide information on the physiological health and the immune status of their members but are also reliable indicators of permanent stress, unlike glucocorticoid levels [37,38]. This is because a stressed individual has increased numbers of circulating neutrophils, while their lymphocytes decrease. Thus, the relative proportions of neutrophils and lymphocytes (N/L ratio) serve as reliable biomarkers of the physiological response, specifically to permanent stress [32,38].

The Cordoba treefrog *Boana cordobae* (Barrio, 1965), formerly described as *Hyla pulchella cordobae*, is an endemic anuran of the provinces of Córdoba and San Luis, Argentina [39,40]. The species status of this treefrog has been recognized by Faivovich et al. in 2004 [41], followed by a reconstruction of its phylogenetic relationships and a generic reassignment [42,43]. These frogs inhabit rocky mountain streams and rivers and are only known to exist at elevations of 808–2310 m a.s.l., in mountainous areas [44]. They exhibit a variable dorsal color pattern. There is a sexual size dimorphism in favor of the females, but males and females attain sexual maturity at the same age of two years [45,46]. Their longevity is five years at lower altitudes and seven years at altitudes higher than 2000 m [46]. From autumn to spring southern hemisphere, i.e., September to March, adults bask on rocks near streams, rivers, and springs. Reproduction is associated with vocal advertisement from the males and takes place in the rock pools of springs, streams, and rivers, where the water flow is slow [47,48,49]. Egg masses are attached to aquatic, mostly submerged vegetation. In cold water, tadpoles need up to 10 months to complete their larval development. The nektonic tadpoles feed on a wide diversity of phytoplankton species, predominantly on diatoms [50,51]. At their metamorphic climax, their total length is 60–90 mm. As the blood markers of tadpoles vary with the fluorite concentration of the streams, they may serve as bioindicators for this pollutant [51,52].

This research was designed to address the still unexplored aspects of life history, the impact of blood parasites on life-history traits, and immune responses to pathogen stress, in *B. cordobae*. We hypothesize that hemoparasites cause metabolic costs for the host, i.e., modify its resource allocation to increase the immune response. Severe consequences for hosts do not seem likely, but detectable variations of morphological, immunological, and demographic host traits are expected to occur [19,21,53]. To evaluate the potential impact of hemoparasites on life-history traits and the immune response, we focused our study on the prevalence and intensity of hemoparasite infection and quantified the corresponding host traits such as size, age, body condition, and leukocyte profile as an indicator of permanent stress.

## 2. Materials and Methods

### 2.1. Frog Collection and Processing

We collected 37 adult frogs (32 males, 5 females) from September 2015 to March 2016 in the central region of the Comechingones Mountains, Córdoba, Argentina (32°50′34″ S, 64°79′30″ W, 1200 m a.s.l.). The sample size was small and sex-biased, likely due to a local population decline as indicated by the fact that a similar survey in 2013/14 during the same season yielded 71 frogs (39 males, 21 females, 11 juveniles) [45]. The study area was a lotic system including several high-altitude streams flowing undisturbed within metamorphic rock. Adults were detected during visual encounter surveys and captured by hand. In situ, we recorded three life-history traits: (1) sex, according to external secondary sexual characters; (2) snout–vent length (SVL), using a digital caliper Mahr (0.01 mm); and (3) body mass, using a Mettler balance (P11N0-1000 g). To determine hematological parameters and hemoparasite prevalence, we collected blood samples from the vena angularis [54] and prepared blood smears. For age determination, we clipped a phalanx of each frog. Before releasing the frogs in situ, antifungal/antibacterial healing agents were added at the puncture site and at the clipped toe to prevent infections.

### 2.2. Microscopical Screening of Blood Smears

In the laboratory, the blood smears were fixed in methanol for 3 min and stained for 10 min with May–Grünwald solution, followed by 10 min in Giemsa solution [55,56]. Slides were examined using a Carl Zeiss trinocular Primo Star (Pack 5) microscope and photographed using an Axiocam ERc 5s digital camera, with ZEN 2.3 lite software. One slide per individual frog was examined. The overall leukocyte titer of each frog was estimated by classifying at least 100 cells as lymphocytes, monocytes, neutrophils, eosinophils, or basophils [35,36]. To obtain an index of immune status, we calculated the proportion of each cell type and the ratio of neutrophils to lymphocytes [34]. Microscopic screening was conducted under equal conditions, i.e., the time spans used for each individual were about the same.

Hemoparasites were classified as morphotypes. We distinguished between extracellular and intraerythrocytic parasites. Extracellular hemoparasites were studied at a magnification of 400×, the intraerythrocytic ones under immersion oil (1000×). Morphological identification of the parasites was based on previous studies [13,20,57,58,59,60,61]. Please note that molecular tools for species identification were not available for this study.

The quantitative descriptors of parasitism were chosen according to Bush et al. [62]: (1) Prevalence, as the proportion of infected individual hosts in relation to the total sample analyzed. (2) Intensity, as the number of extracellular hemoparasites per infected host in 100 microscope fields (magnification 400×). For intraerythrocytic hemoparasites, the intensity was calculated as the percentage of infected erythrocytes per host out of 1000 cells examined. (3) Mean intensity, as the arithmetic mean of all individual densities.

### 2.3. Skeletochronological Age Determination

The demographic life-history trait of age was assessed using the standard protocol of skeletochronology [63,64], as follows: (1) fixation in formaldehyde 4% (at least 12 h); (2) decalcification of bones (10% formic acid, 24 h); (3) paraffin embedding; (4) cross-sectioning of the diaphysis 10–12 μm using a rotary microtome (Leica^®^ RM2125 RTS); (5) staining with Ehrlich’s hematoxylin (2 min); (6) light microscopic count of the number of lines of arrested growth (LAGs) using a light microscope (Zeiss Axiophot-Axiolab, 100×); (7) documenting the most informative cross sections with a digital camera (Axiocam ERc 5s, software ZEN 2.3 lite. 4.3). Age was estimated by examining 10 cross sections per individual and counting the number of LAGs in the periosteal part of the bone, performed independently by two observers (M.B. and M.O.). We tested for potential endosteal resorption following the protocol outlined by Lai et al. [65]. Age was defined as the number of LAGs, plus one to account for the year following the last LAG formation during winter. We refrained from estimating growth parameters based on the von Bertalanffy equation [66] because the low number of parasite-free individuals, and the male bias, did not allow for reliable estimates.

### 2.4. Data Analysis

Descriptive statistics are given as the mean ± standard error. The Kolmogorov–Smirnov test was used to compare the shape of distributions. Analysis of Variance (ANOVA) was used to compare statistically sex-specific parasite loads and levels of infections with parasite-free controls based on log10-normalized data. As the age, SVL, and condition data of the males were normally distributed, we used untransformed data to compare the life-history traits of parasite-free and infected individuals in Analyses of Variance (ANOVA). In additional analyses of covariance (ANCOVA), we considered the usually correlated SVL and age parameters as covariables. We fitted regression models (model selection based on maximum R^2^) to the relationships between SVL and age, and SVL and body mass, respectively. The body condition of an individual was calculated as the studentized residual of the SVL–mass relationship using a multiplicative model, as follows: ln(mass) = a + b × ln(SVL), where a = intercept and b = slope (residual index, as detailed in [67]). The significance level used was *p* < 0.05. Tests were performed using the statistical package STAGRAPHICS Centurion XVIII (Statpoint, Inc., Warrenton, VA, USA, 2018).

## 3. Results

**Figure 1:**
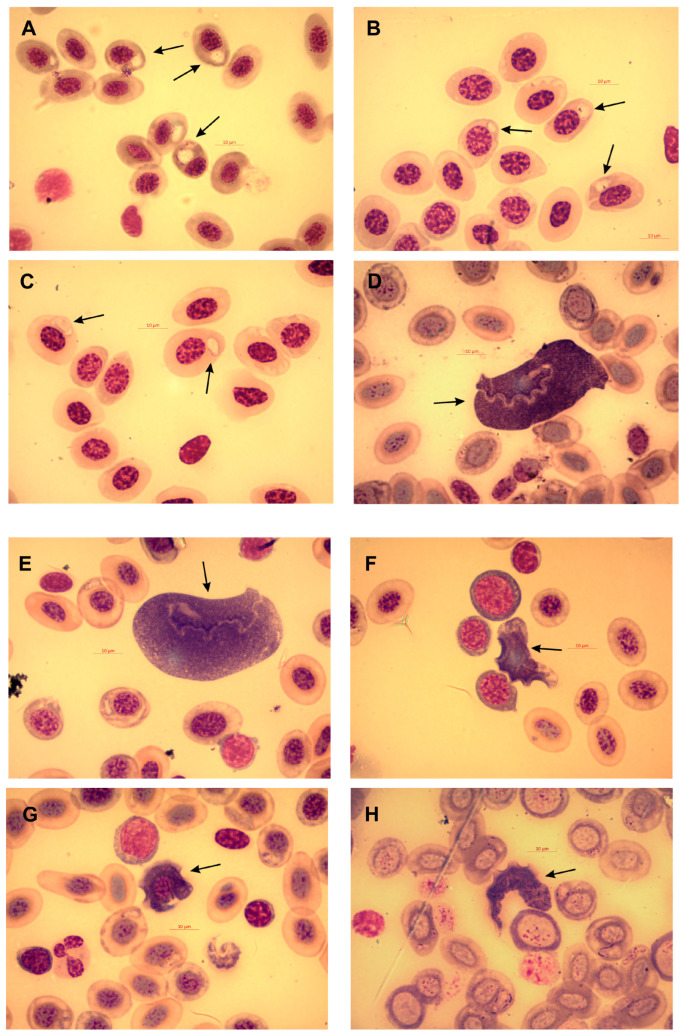
Hemoparasites detected in blood smears of Cordoba treefrog (*Boana cordobae*). (**A**–**C**) *Dactylosoma* spec.; (**D**–**H**) *Trypanosoma* spp. (**D**,**E**) Morphotype “A” cells with rounded or elliptical trypomastigotes; (**F**,**G**) Morphotype “B” elongated trypomastigotes with pointed ends; (**H**) Morphotype “C” elongated trypomastigotes with pointed ends. Arrows indicate hemoparasites. Scale bar is 10 μm.

Most of the *B. cordobae* (n = 30, 81.1%) that we collected were infected (25 males, 5 females) by hemoparasites (Figure 1). Both extracellular and intraerythrocytic hemoparasites were detected in twenty-three individuals (twenty males, three females), exclusively extracellular parasites were detected in two males and two females, and exclusively intraerythrocytic ones were detected in three males. None of the frogs infected with hemoparasites showed external morphological signs of disease, or deviated in behavior from parasite-free individuals.

### 3.1. Taxonomic Assessment and Features of Hemoparasites

The intraerythrocytic parasites were morphologically identified as hemogregarines belonging to the genus *Dactylosoma* (Apicomplexa, Dactylosomatidae) (Figure 1A–C). The light microscopic examination did not allow for species identification, and molecular assessment was not available. All extracellular blood parasites detected were trypanosomes (Euglenozoa, Trypanosomatidae), standing out among the blood cells because of their large size and characteristic morphology. We found classical fusiform trypomastigotes as well as rounded, oval, fan-shaped, leaf-shaped, or irregular cells, with or without a free flagellum. The trypanosomes included three distinct morphotypes: rounded or elliptical cells represent morphotype “A” (Figure 1D,E), rounded trypomastigotes with pointed ends represent morphotype “B” (Figure 1F,G), and elongated trypomastigotes with pointed ends represent morphotype “C” (Figure 1H). The prevalence, intensity, and average intensity of *Dactylosoma* and *Trypanosoma* hemoparasites did not differ significantly between males and females (Table 1).

### 3.2. Leukocyte Profiles

**Figure 2:**
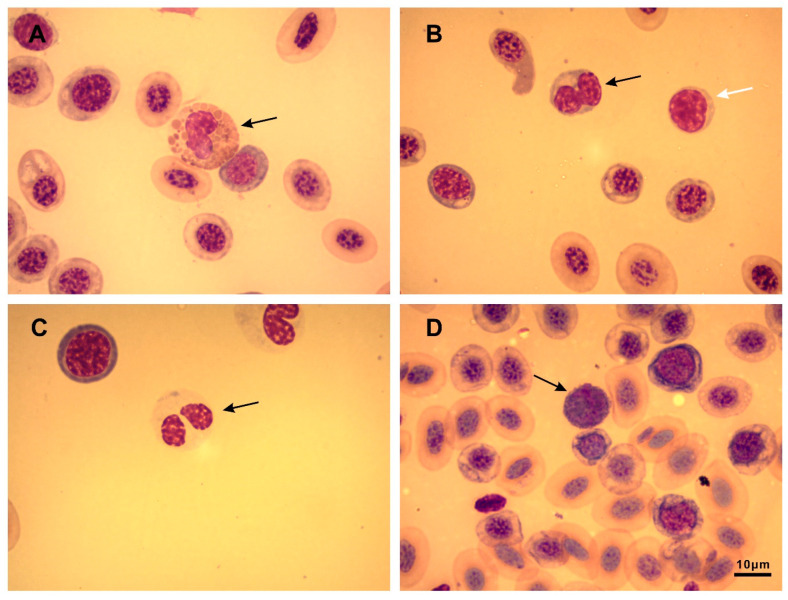
Different types of leukocytes in blood smears of Cordoba treefrogs (*Boana cordobae*). (**A**) Eosinophil (arrow); (**B**) lymphocyte (black arrow), monocyte (white arrow); (**C**) neutrophil (arrow); (**D**) basophil (arrow). Scale bar is10 μm.

For this analysis, we pooled data of the males and females. Lymphocytes were the dominant group of leukocytes, followed by neutrophils (Figure 2, Table 2). Parasite-free frogs tended to have more lymphocytes and less neutrophils and basophils, but this difference was not statistically significant. The average N/L ratio of the parasite-free frogs was only about half of that of the infected ones, but again, this difference was not statistically significant.

### 3.3. Influence of Hemoparasites on Life-History Traits

**Figure 3:**
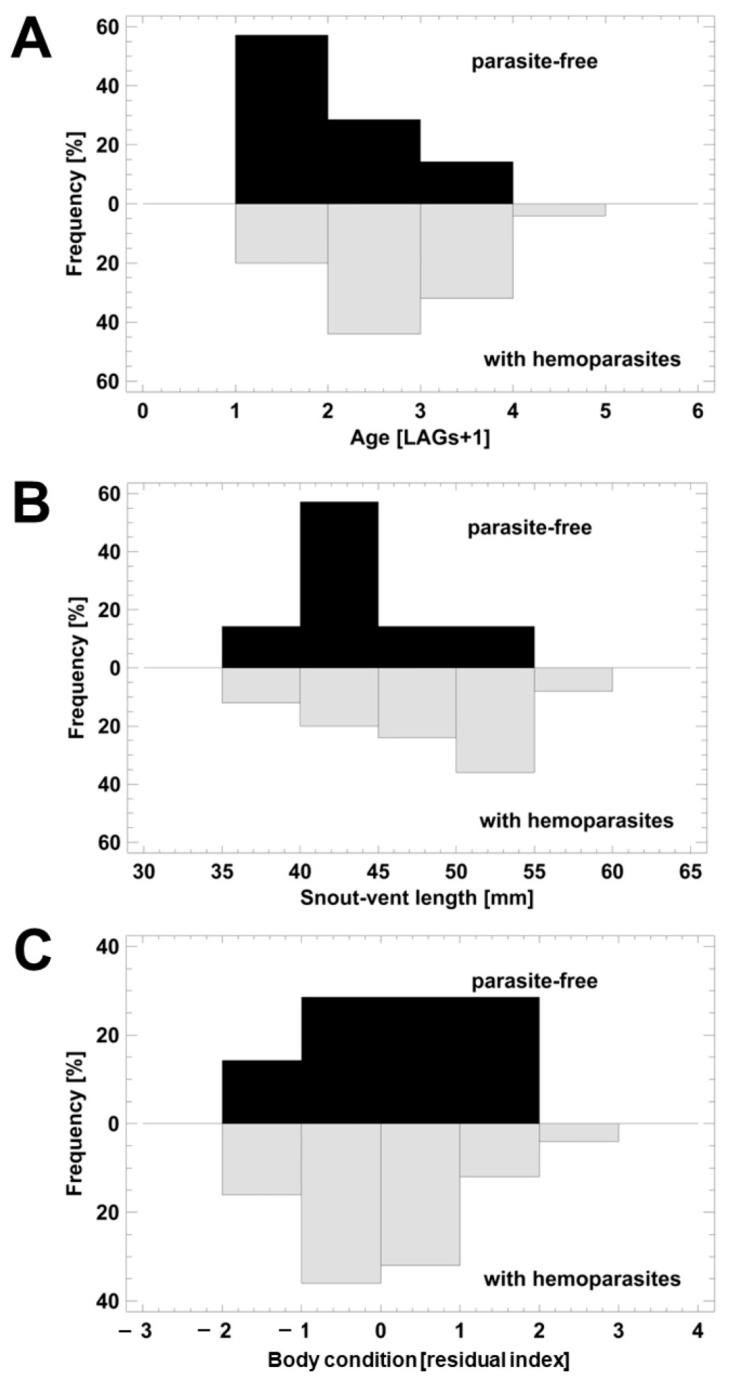
Life-history traits of parasite-free and infected Cordoba treefrogs (*Boana cordobae*). (**A**) Age; (**B**) SVL; (**C**) body condition. Details on statistical comparison are in the text.

Because of the low number of females available and the fact that all were infected, we limited the analysis of the impact of parasitism on life-history traits to exclusively males. Their age ranged between 2 and 5 years (Figure 3A). Age did not differ significantly between the infected and parasite-free males (ANOVA, F_1,30_ = 3.49; *p* = 0.0798). Still, the shape of the age distribution was biased significantly toward younger individuals in parasite-free frogs (Kolmogorov–Smirnov test, *p* = 0.01777; Figure 3A). Snout–vent length varied between 38 and 58 mm. SVLs of the parasite-free frogs were significantly smaller than those of the infected ones, if uncorrected for age (ANOVA, F_1,3_ = 4.77; *p* = 0.0369; Figure 3B). This statistical significance disappeared when considering age as a covariable (ANCOVA, F_1,29_ = 1.81; *p* = 0.1886). As indicated by the ANCOVA, SVL was significantly correlated to age (double-reciprocal regression model, R^2^ = 36.6%; F_1,30_ = 17.30; *p* = 0.0002). Body condition (as a residual index) did not differ between the two groups (ANOVA, F_1,30_ = 0.34; *p* = 0.5660; Figure 3C). Adjusting the body condition results using age and SVL as covariables did not change the absence of this statistical significance (ANCOVA, F_1,28_ = 0.35; *p* = 0.5584).

## 4. Discussion

Our study provides the first evidence that the endemic treefrog *Boana cordobae* is a new host for hemogregarines of the genus *Dactylosoma* and flagellates of the genus *Trypanosoma*. An extraordinary high proportion of 81.1% of wild free-living *B. cordobae* were found to carry at least one type of hemoparasite. In Argentina, limited research has been conducted on anuran amphibians affected by hemoparasites. To our knowledge, we report the first *Dactylosoma* ever detected in Argentina. Cabagna-Zenkluzen et al. [68] presented in 2009 the first records of hemoparasites (*Trypanosoma*, microfilariae) in leptodactylid and hylid frogs from Argentina. Trypanosomes were found in *Leptodactylus chaquensis* (5–22% prevalence), *L. ocellatus* (9%), and *Trachycephalus venulosus* (17%), i.e., in prevalences far below the levels reported here. The prevalence in anurans infected with trypanosomes was also lower in Brazil (10% in *Boana multifasciata,* 43% in *B. geographica*), in Panama (40% in male and 7% in female *Engystomops pustulosus*), in South Africa (11%), and in Thailand (34% in *Hoplobatrachus rugulosus*) [15,27,69,70]. This geographical review on trypanosome-infected anurans suggests that their prevalence in *B. cordobae* is exceptionally high. With respect to *Dactylosoma* infections, its 70% prevalence in *B. cordobae* also contrasts with the reported prevalences of 6% in *Rhinella major*, 16% in *Leptodactylus ocellatus/labyrinthicus* in Brazil, and 28% of *Ptychadena anchietae* in South Africa [15,29,71,72]. Further investigations will be needed to elucidate the reasons for the remarkably high prevalence of hemoparasites in *B. cordobae*.

Multiple infections with high pathogen intensities seem to be the trend in *B. cordobae*, as has been observed in many Palearctic and Neotropical anuran hosts as well [13,61,68,69,70]. Still, external manifestations of pathogenic impacts were absent, as they are in other anurans carrying hemoparasites, but inflammatory lesions, predominantly in the liver, have been reported in other frog hosts infected with hemogregarines [12,70].

An activation of the cellular immune system in response to *Rickettsia* hemoparasites is indicated by changes in the leukocyte profile of parasitized salamander individuals [34]. The main response seemed to be an increase in the total leukocyte number, whereas the single fractions did not show significant intergroup differences, except for eosinophils [34]. Similarly, we observed an increase in eosinophils to levels more than twice as high in parasitized frogs compared to those of the parasite-free controls; however, this was not statistically significant. This lack of significance may be an effect of the small sample size or, alternatively, may indicate that there is no adaptive response.

In vertebrates, an immunological stress indicator is the ratio between their neutrophil and lymphocyte densities, as during infections, their neutrophils increase and their lymphocytes decrease [34,73]. Neutrophils are known to phagocytize and target foreign particles and microbes and they increase numerically in response to injuries or infections [73]. Yet, recent studies on parasitized amphibians have found only minor changes in their leukocyte profile [34,74]. Our study is in line with the observation that trypanosomes and hemogregarines do not cause significant changes in the N/L ratio, as we noticed only a slight and insignificant increase from 0.03 to 0.06 in *B. cordobae*.

A high prevalence and intensity of hemoparasites may negatively affect the life history and fitness of a reptile host by reducing its capability to transport oxygen and changing its allocation of resources [75,76,77], whereas similar effects in amphibian hosts have not been reported so far. In *B. cordobae*, we could not confirm any difference in the life-history traits we studied between infected and parasite-free individuals, suggesting that parasitism could potentially exert a limited or negligible impact on the host’s fitness. At first glance, the surprising fact that the infected frogs were significantly larger than the parasite-free controls seems to be due to the dominance of young and subsequently small individuals in the controls (this size–age relationship has been established in a previous study [32]). Thus, infection seems to occur at the post-metamorphic stage of life, in agreement with similar observations of *Lithobates clamitans* and *L. catesbeianus* [17]. The stage of life in which individuals are most often infected might be the first breeding period of an individual and the infection may remain for life, like in *Notophthalmus viridescens* [78]. Potential vectors of hemoparasites are leeches and hematophagous dipterans [79,80,81]. Mosquitoes (*Uranotaenia* spp.) and frog-biting midges (*Corethrella* spp.) are known to be attracted by anuran advertisement calls and to transmit a wide range of hemoparasites [26,27,82]. In this case, the prevalence of infection is sex-biased toward males, as shown in *Engystomops pustulosus* and in *Dryophytes cinereus* [25,26,27]. We do not know the local vectors responsible for transmitting parasites to *B. cordobae* hosts, but the fact that all females were infected does not suggest a role of vocalization in vector attraction.

## 5. Conclusions

In conclusion, we have demonstrated for the first time that moderate to high densities of hemoparasites do not affect significantly the cellular immune response of *B. cordobae* hosts or any of the life-history traits studied. Infection of the frogs seems to occur usually following sexual maturation and seems to persist until death, but its vector and mode of transmission remain to be identified. Further studies are needed in which other measures of the host’s fitness are considered, such as growth rate, feeding rate, locomotor performance, reproductive status, and reproductive output, to understand this complex host–parasite relationship.

## Figures and Tables

**Table 1:** Quantitative descriptors of hemoparasites infecting *B. cordobae*. Statistical comparison of the mean intensity between males and females refers to an ANOVA with log10-normalized data.

Hemoparasites	Prevalence (%)	Intensity (Range)	Mean Intensity (n ± SE)	ANOVA
Intraerythrocytic (*Dactylosoma*)				
Males	72	1–40	10.5 ± 2.3	*p* = 0.9841
Female	60	1–22	8.7 ± 6.7	
All adults	70	1–40	10.3 ± 2.2	
Extracellular (*Trypanosoma*)				
Males	69	1–50	10.0 ± 2.5	*p* = 0.0907
Female	100	2–28	8.6 ± 5.0	
All adults	73	1–50	9.8 ± 2.2	

**Table 2:** Leukocyte profiles measured in parasite-free *B. cordobae*, and in those infected with exclusively *Dactylosoma* (D) or *Trypanosoma* spp. (T), and in those with both simultaneously (D + T). n: sample size; N/L ratio: neutrophils/lymphocytes ratio. Statistical comparison of groups (ANOVA) refers to log10-normalized data.

Leukocytes [%]	Parasite-Free Individuals (n = 7)	Infected Individuals (D) (n = 3)	Infected Individuals (T) (n = 4)	Infected Individuals (D + T) (n = 23)	ANOVA
Lymphocytes	94.1	89.4	89.3	89.9	*p* = 0.5994
Neutrophils	3.3	6.6	5.6	5.2	*p* = 0.5269
Eosinophils	0.9	2.3	2.0	2.6	*p* = 0.4838
Basophils	0.6	0.3	2.7	1.2	*p* = 0.0288
Monocytes	1.1	1.3	0.5	0.3	*p* = 0.6328
N/L ratio	0.036	0.063	0.076	0.066	*p* = 0.8610

## Data Availability

All data used for this manuscript are given in the text.
